# Determining quantitative targets for public funding of tuberculosis research and development

**DOI:** 10.1186/1478-4505-11-10

**Published:** 2013-03-08

**Authors:** David R Walwyn

**Affiliations:** 1 Department of Engineering and Technology Management, Graduate School of Technology Management, University of Pretoria, Pretoria 0002, South Africa

## Abstract

South Africa’s expenditure on tuberculosis (TB) research and development (R&D) is insignificant relative to both its disease burden and the expenditure of some comparator countries with a minimal TB incidence. In 2010, the country had the second highest TB incidence rate in the world (796 per 100,000 population), and the third highest number of new TB cases (490,000 or 6% of the global total). Although it has a large TB treatment program (about $588 million per year), TB R&D funding is small both in absolute terms and relative to its total R&D expenditure. Given the risk and the high cost associated with drug discovery R&D, such neglect may make strategic sense. However in this analysis it is shown that TB R&D presents a unique opportunity to the national treasuries of all high-burden countries. Using two separate estimation methods (global justice and return on investment), it is concluded that most countries, including South Africa, are under-investing in TB R&D. Specific investment targets for a range of countries, particularly in areas of applied research, are developed. This work supports the outcome of the World Health Organization’s Consultative Expert Working Group on Research and Development: Financing and Coordination, which has called for “a process leading to the negotiation of a binding agreement on R&D relevant to the health needs of developing countries”.

## Introduction

Tuberculosis (TB) is a bacterial disease that is caused by pathogenic bacteria *Mycobacterium tuberculosis* (Mtb). Although the number of new TB cases has been falling since 2006, the disease is still a global epidemic. According to the World Health Organization (WHO) [1], in 2011 there were 8.8 million incident cases of TB, 1.1 million deaths from TB among HIV-negative people, 0.35 million deaths from HIV-associated TB and almost 10 million children orphaned as a result of parental deaths caused by TB.

South Africa is facing a massive TB epidemic [2], which is threatening its social and economic well-being through both the direct cost of treatment and the loss of productive economic activity. Already the cost of treating TB (medicines and hospitalization) is in excess of $588 million per annum [3,4] (further details on the TB treatment budget and the burden of disease are given in Additional file 1) and the estimated loss to the GDP is about $3.06 billion per annum [5]. The HIV epidemic is amplifying the problem; not only is this epidemic directly increasing the number of TB patients, but it is also now recommended that HIV patients receive isoniazid preventative therapy (IPT), thereby adding a further approximately 1.5 million patients to the 460,000 TB patients who require therapy or prophylaxis each year (according to the WHO [1], in 2010 only 124,049 HIV positive patients received IPT).

In response to this situation, the South African National Department of Health has adopted the Negotiated Service Delivery Agreement (NSDA) [6], which contains a number of TB-specific objectives including a decrease in the burden of disease from Mtb, an increase in the TB cure rate from 64% to 85%, and an increase in the TB treatment completion rate. However, the achievement of the NSDA goals will require more than the wider utilization, and hence additional expenditure, on diagnosis and treatment. Higher completion rates in particular will only be possible with new drugs.

Under the present regimens, treatment for drug-sensitive TB requires 6 months of medication and for drug-resistant TB at least 18 months, making patient adherence and regimen completion extremely difficult. These extended timelines are in sharp contrast to both the intrinsic killing rates of these drugs in bacterial cultures and the action of standard antibiotics, and it has been hypothesized that upon drug exposure Mtb undergoes metabolic changes rendering it immune to the bactericidal activity of TB inhibitors [7]. New therapies that are short-acting, that are effective against all forms of Mtb (latent, actively replicating and drug-resistant forms) and that prevent the emergence of Mtb granulomas, are urgently needed to help combat the disease [7-10].

### TB drug discovery requires public investment

Unfortunately, the development of new drugs and regimens is expensive, time consuming and of uncertain outcome. “How long” and “how expensive” remain disputed quantities, and depend on a number of factors including assumptions about success rates and patient cohort sizes. However, the long time scales, large investments and low rates of return make public investment essential.

Detailed support for this statement has been provided in the Additional file 1. The risk-adjusted net present value approach has been used in order to estimate total development costs; the latter is a correction based on the estimated success rate of the project at different stages of development [11]. This calculation results in the estimation of the parameter risk-adjusted net present value (rNPV), which is used as the primary indicator of project profitability.

**Figure 1:**
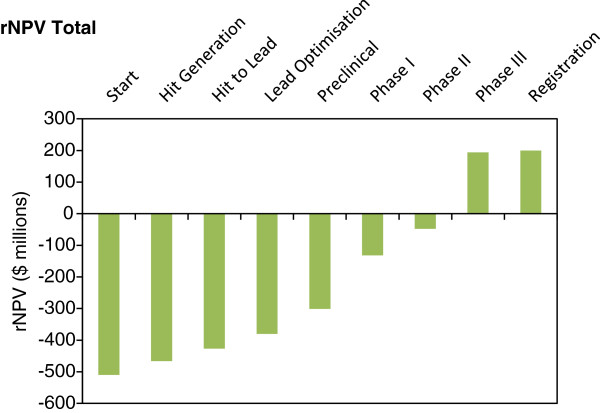
Project rNPV for development of first-line TB drug.

Based on a range of values for the above parameters, the rNPV for a novel TB drug development project has been calculated (Figure 1).

It is clear from Figure 1 that the economics of TB drug development, production and sale are insufficient to attract the involvement of a private investor prior to Phase III (the product has a positive rNPV only at the end of Phase III). This conclusion is the consequence of the high rate of failure in drug discovery and the low TB market size, and contradicts previously published data [12,13]. Based on a detailed assessment of a wide cross-section of data [14-17], it is the author’s opinion that the TB Alliance report has overestimated the potential return on investment due to several highly optimistic assumptions including success rate, market share, margin on sales and net development costs.

Given the negative return prior to end of Phase III, it is unlikely that the standard model in which drug candidates are licensed to a private company (development partner) in the preclinical stage will apply to TB drug discovery. To a large extent, this conclusion is confirmed by the published data on TB research. In 2010, 80% of the total TB R&D expenditure came from public sector or philanthropic funding [18,19] and many of the Phase II/III clinical trials are being supported by public research institutions, development agencies or product development partnerships, the latter receiving much of their funding from philanthropic organizations [19]. In the same year, there were only 4 private companies in the top 15 TB R&D funders, and of the $127 million spent on basic TB research, only 5% came from a private company [19]. It is clear that without support for TB drug development from public funds, we will not have new TB treatment drugs or regimens.

The analysis in this section applies specifically to the case of TB drug development, and will not necessarily apply to research on other types of products or services such as diagnostics or operations research. However the conclusions do present a compelling case for significant public sector support for TB drug development, without which such new drugs will not be developed or become available. The returns for a private company are simply inadequate to attract long term and significant investment in the development of new TB drugs.

A different conclusion can be reached, on the other hand, for the public sector, which forms the dominant customer for TB treatment. New technologies and products should result in significant savings to treatment costs; despite this potential return, TB R&D has failed historically to attract sufficient funding from either the public or the private sector and places the illness in the category of a neglected disease. Further details on R&D funding for the latter now follow.

### Neglected disease R&D

In the more general discussion of neglected disease R&D, it is useful to distinguish between two disease categories, namely Type II which is defined as “incident in both rich and poor countries, but with a substantial proportion of cases in poor countries” (TB is an example of a Type II disease) and Type III, which is defined as “overwhelmingly or exclusively incident in the developing countries” (such as river blindness and sleeping sickness). For a long period, neglected or poverty-related disease R&D was “at a virtual standstill” [20]. Following various civil society initiatives, the situation over the past 10 years has improved, with funding for R&D on Type II neglected diseases increasing substantially. Examples of these initiatives include the establishment of several open models for innovation such as the Medicines Patent Pool, Open Source Drug Discovery and World Intellectual Property Organization Re:Search database; and the introduction of new incentive mechanisms to address market failures such as the US Food and Drug Administration priority review voucher and the US Patent and Trademark Office‘s ’Patents for Humanity’ initiative.

The situation for Type III diseases remains largely neglected with authors of the G-FINDER survey noting that nearly 80% of the total neglected disease R&D funding is spent on TB, HIV and malaria (all Type II), despite the higher disease impact of several Type III diseases [21]. Moreover there are ongoing concerns about sustainability of even Type II funding with calls for WHO member states “to begin a process leading to the negotiation of a binding agreement on R&D relevant to the health needs of developing countries” [22]. The concerns are focused on two major limitations in the present situation, namely that neglected disease R&D is heavily reliant on a few donors (the Bill & Melinda Gates Foundation in particular), and that the priorities for health R&D are determined by these funders, rather than through a well-coordinated, global R&D framework [20].

The report of the WHO’s Consultative Expert Working Group on R&D (CEWG) recommends that “a binding instrument on R&D is necessary to secure appropriate funding and coordination to promote R&D needed to address the diseases that disproportionately affect developing countries and which constitute a common global responsibility” [22]. This report also proposes for all countries a target of 0.01% of the GDP for government-funded R&D devoted to the health needs of developing countries, and specifically for developing countries with ‘potential’ research capacity, a commitment of 0.05 to 0.1% of GDP [22].

**Figure 2:**
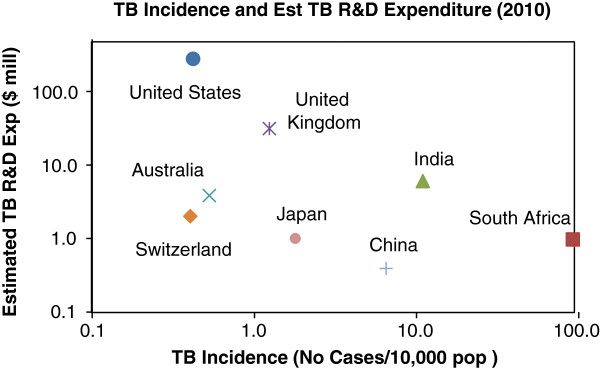
**Estimated expenditure on TB R&D relative to TB disease burden.** Sources: WHO [1] and G-FINDER [24]. Note that the TB R&D expenditure is a factored estimate based on the total reported neglected disease R&D expenditure for each country; actual values are unfortunately not available.

At the core of the debate about funding and priorities is the role of governments within national states and public health systems. To date, governments of countries with high endemic levels of poverty-related diseases (typically low-income countries) have contributed disproportionally little towards neglected disease R&D, including TB (Figure 2). Moreover, health research in these countries also lacks an overall framework and a set of well-defined national research priorities [23]. The result is a double deficit, with both the total quantity of health research being inadequate and the content of what little research is being undertaken, being of limited relevance to national needs. It is also evident from Figure 2 that donors in the United States and the United Kingdom together account for more than 95% of TB R&D, a surprising observation given the low TB incidence of these two countries. The stark reality is that most global TB research is being undertaken by two high-income countries, whose objectives may not overlap with the priorities of low-income countries.

**Figure 3:**
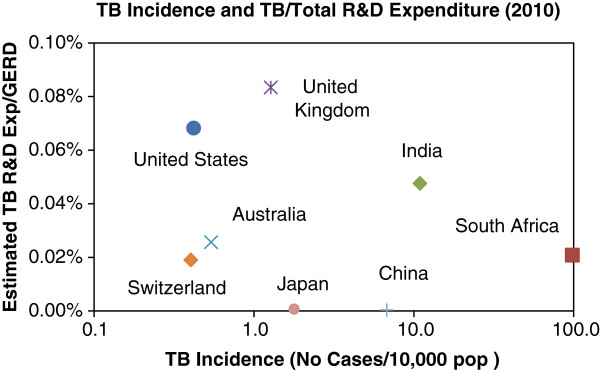
**Estimated TB R&D expenditure relative to GERD and TB burden.** Sources: WHO [1], G-FINDER [24] and OECD [28].

Few would argue with the statement that the right to health is a human right, and that governments have an obligation to protect and develop the health of their own citizens [25]. It can also be argued that in terms of global justice principles, high-income countries have an obligation to the global poor [26,27], which may help to explain the data in Figure 2. However, the efforts of high-income countries to support TB R&D does not mean that middle- or low-income countries are relieved from their obligations towards their own citizens, and that adequate support for health research should not be a priority in the allocation of national funds within such countries. For instance countries such as India, China and South Africa, which have a high TB incidence, also have a comparatively sizeable gross expenditure on R&D (GERD) and could afford to spend more on TB research (Figure 3). The present allocations of less than 0.05% of GERD and lower to TB R&D seems hardly appropriate given the urgency of the problem and previously stated principles of global justice and health as a human right.

Notwithstanding the moral and legal arguments, the perceived correlation shown in Figure 3 is further counterintuitive considering the obvious benefits to a high-burden country of developing improved TB treatment. From the introduction, it is clear that a high TB burden equates to a high social and economic cost, particularly to the national treasury since most countries cover TB treatment within their respective public health programs. It could be expected that the development of new TB therapies in high incidence countries, as a means of reducing costs in the longer term, would be highly attractive. The reasons for this ongoing neglect of health research remain obscure unless one accepts the argument that national treasuries as a general rule shun health research on the basis that such investments carry high risk with limited hope of reward.

Indeed such a position is partly true; as noted earlier, drug discovery in particular is expensive and high risk, and such an undertaking can perhaps only happen in developed countries with the appropriate supporting infrastructure and required expertise. However risk and return is only a part of the explanation; there is a great deal more to health research than drug discovery. For instance, the CEWG report recognizes the importance of other areas, including research on novel vaccines, diagnostics, health systems, operational and implementation issues, monitoring and evaluation, and health-related policy issues [22]. Some of these areas have the potential for high impact and low risk, especially operational and implementation issues.

The CEWG report also reviews the history of targets for national financing of health and health R&D, including the Abuja target of 15% of GDP on health financing [29] and the Commission on Health Research and Development (CHRD) target of 2% of national health expenditure on health R&D [30], equivalent to 0.3% of GDP. It is noted that most developing countries have failed to meet either the Abuja or the CHRD targets, and that more direct and proactive means are required to stimulate global TB R&D. This failure has been acknowledged by South Africa in the proceedings of the 2011 National Health Research Summit [31], which noted that “there is inadequate funding of health research by the Government of South Africa, especially by the Department of Health”, with the department investing about 0.37% ($49 million) of its 2011/2012 health budget ($13.2 billion) in health R&D, a shortfall of $216 million *vs.* the 2% target adopted by the Health Research Policy in South Africa in 2001.

It is not clear how these targets were determined, or how they relate to priorities and affordability. In the remainder of this article, the determination of more justifiable and realistic targets for health R&D, using the case study of TB research in South Africa, is defined and discussed in more detail. A Global Justice Index, based on GDP/capita, and a Return on Investment (RoI) Factor are developed and then combined to give a target for national TB R&D expenditure for South Africa and other countries. Although the specific example of TB is used as a case study, it is noted that the same methodology could be applied equally to the broader area of neglected diseases.

### Computing the optimal TB R&D expenditure

Computing the optimal or justifiable TB R&D expenditure for any country requires an assessment of the competing priorities and the affordability of/return on investment from such a public-financed program. In developing a methodology for determining the appropriate expenditure target, the following factors have been considered:

•The TB disease burden as measured by the incidence rate per 10,000 population;

•The national R&D budget and the GDP per capita;

•The size of the national treatment program (which can be considered as the capacity for budget savings).

These factors are used to compute the Global Justice Index and the RoI Factor, which are now discussed in more detail.

#### Global justice index

**Figure 4:**
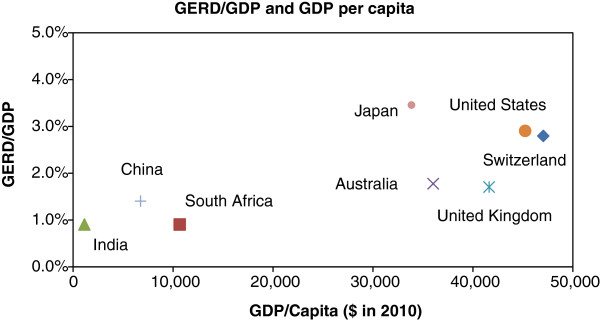
**National R&D intensity correlates with GDP per capita.** Source: OECD [28].

It is well known that national R&D intensity, calculated as the ratio of GERD to gross GDP, correlates with GDP per capita [32,33] (Figure 4). The interpretation of this correlation has always been contested, with ongoing debate about whether high GDP per capita is a consequence or a cause of a high GERD/GDP ratio [34]. Undoubtedly the two variables are interlinked and will influence each other in a complex, sometimes unpredictable manner. Nevertheless, GDP per capita is certainly indicative of a country’s capacity to support research and development, and high levels of income are typically associated with higher research expenditure.

The allocation of research funds to specific areas and research projects is almost entirely a discretionary process. Given a pre-determined budget, allocation decisions are made on the basis of strategic alignment, political factors and a host of other considerations such as available resources, expected impact and infrastructure. All nations, and organizations within these countries, which have an R&D budget, have as a consequence the opportunity to invest in TB research.

This discretionary nature of research allocation decisions lies at the core of resolutions such as the Abuja declaration [29] and the CHRD target. It seems reasonable to propose that nations which have discretion about how public funds are allocated, should set aside a specific budget for health research, and for the purposes of this analysis, to TB research, based on the principles of global justice.

The question then becomes “what is a reasonable allocation?” given the previously outlined obligations of developed or wealthy nations to the global poor. The CHRD target of 2% of national health expenditure for health research is certainly a beginning point, but the value is empirically stated and not theoretically derived. Using data from the G-FINDER [24] and TAG [19] reports, regression analysis has been used to derive a correlation between GDP per capita and TB R&D. The results of the regression follow:

For GDP/capita < 5,000; TB R&D = 0

For 5,000 < GDP/capita < 60,000; TB R&D = 4.2 × 10^-10^ × (GDP/capita)^2^ – 1.6^-06^ × (GDP/capita)

For GDP/capita > 60,000; TB R&D = 1.42

(where TB R&D is expressed as $/capita)

The source and calculated data are shown in Table 1 (including the RoI Factor, as discussed later).

**Table 1:** Proposed targets for TB R&D expenditure based on GDP and incidence

**Country**	**GDP/Capita (USD million/ capita)**	**Present TB R&D (USD million)**	**Proposed target TB R&D (USD million)**		
			**Global justice index**	**RoI factor**	**Total**
United States	47,040	278	265	29.5	294
Switzerland	45,236	2	6.3	0.9	7.2
United Kingdom	36,030	33	30.2	6.5	36.8
Australia	41,622	4	14.5	1.2	15.8
Japan	33,873	1	54.3	51.5	106
South Africa	10,676	3	1.5	90.0	91.5
China	6,746	0.40	11.2	117.6	129
India	1,143	6	1.2	57.4	58.6

Source: G-FINDER [24] and OECD [28].

It is noted that the regression is based on the two propositions of national responsibility in terms of global justice and affordability based on national wealth. The coefficients are significantly influenced by the two countries of United Kingdom and USA, both of which are major funders of TB research. It is a non-linear function, which has an upper and lower limit, with the lower limit being $5,000 GDP per capita. In essence the correlation enables the quantification of the extent of a country’s obligation to support global TB research and has been labeled as the Global Justice Index.

#### Return on investment (RoI) factor

An alternative and additional argument to the proposal that high income countries should fund TB R&D based on a global justice principles, is that high-burden countries should act similarly since these countries have the most to gain in terms of savings to TB programs and better utilization of taxation revenues.

In many countries the public sector is the main financier of TB treatment (in South Africa, 97% of incident cases are treated in the public sector) [35]. In this capacity, national treasuries will benefit from improved TB treatment in the following respects:

•Reduction in treatment time and number of drugs: with a new drug, it is likely that TB treatment time can be reduced from 6 to 2 months, and the number of drugs from 4 to 2. Most recent regimens currently being tested contain fewer drugs and are effective in shorter time periods [7-10]. In South Africa, it is calculated that the net cost savings to the National Treasury will be about $82 million per annum; the latter figure is based on a reduction in hospitalization costs for MDR-TB and lower drug costs (presently $207 million and $14 million respectively).

•Improved treatment outcomes: the present re-treatment rate is about 15% of the total patients treated per annum (60,580 patients) [1]. It is assumed that the re-treatment rate will fall to less than 1% with an improved regimen, thereby saving a further $89 million per annum (this assumption is considered reasonable given the cure rates for analogous bacterial infections).

•Job creation: the local manufacture, marketing and distribution of a new drug will create jobs in the chemical industry, the pharmaceutical industry and the healthcare industry. It is not possible to specify how many jobs and in which sector due to the uncertainty of the commercialization route.

•Economic growth: the anticipated product revenue of a new TB drug in South Africa alone will be about $5 million per annum, which will replace about $14 million of imported pharmaceuticals and will reduce the total per patient treatment cost from about $33 to $14 per patient course. Similar figures could be expected for other countries.

These savings and benefits have already been mentioned from a qualitative perspective. However, quantification of these benefits is more difficult to define. In this analysis, the following assumptions have been made:

**Figure 5:**
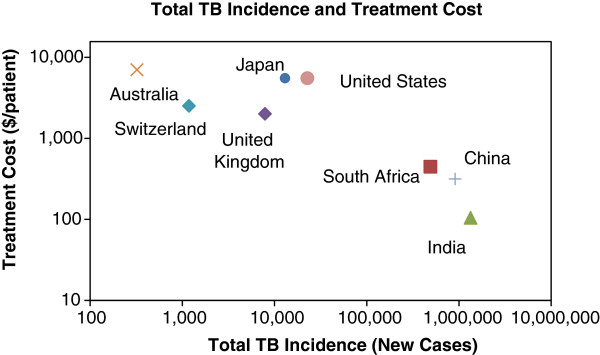
**TB incidence and treatment cost per patient (2010).** Source: WHO [1].

•Program budget: before any cost savings can be estimated, it is important to value the cost of the existing program. This is calculated from the WHO data on incidence and treatment cost per TB patient (Figure 5).

•Extent of annual savings: the value of a R&D project from the perspective of a national treasury can be considered as the net present value of the savings over the expected duration of the innovation. Estimating the annual savings over a range of project types, technologies and interventions is clearly unrealistic. However, it is noted that managers of public health programs use a figure of about 15% as a guide in the evaluation of a proposed new program [personal communication from Dr Anban Pillay of the National Department of Health, South Africa]. Below this value, the cost of implementation is considered to exceed the extent of the savings, such costs being typically associated with activities such as the revision of guidelines, training of personnel and amendments to tendering procedures. As a conservative estimate, it is therefore assumed that the R&D outcomes will at a minimum achieve the 15% hurdle rate.

In many cases, the annual savings will be higher. For instance, a new TB drug which will reduce treatment time from 6 months to 2 months, number of drugs from 4 to 2, and the daily drug dose from 1,625 mg (pyrazinamide) to 200 mg (delamanid) could decrease the TB treatment cost per patient from in excess of $30 to $20.

•rNPV of future savings: the risk adjusted NPV of the future savings depends on the expected duration of the savings, the perceived risk and the discount rate. The latter has been assumed previously at 8% and the average expected duration as 15 years. The perceived risk will be dependent on the project type; for instance a drug discovery project will have a higher risk than a new diagnostics project. Nevertheless, across a whole portfolio of health R&D projects, the average risk factor, based on the weighted average success rates (Additional file 1: Table S4) will be about 30%.

**Figure 6:**
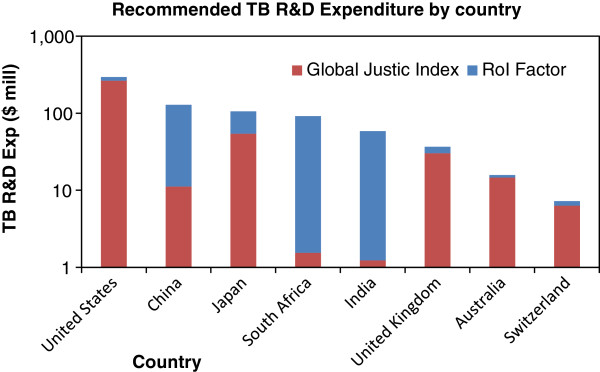
Recommended TB R&D expenditure based on global justice index and RoI factor.

**Figure 7:**
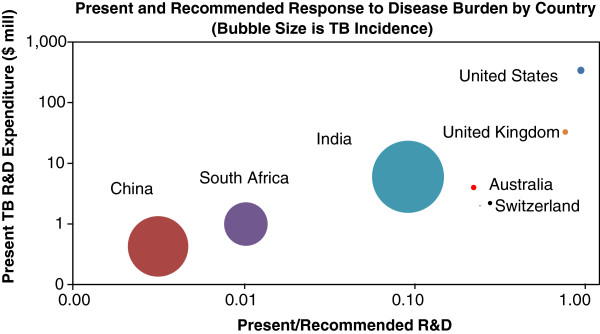
**Actual and recommended TB R&D expenditure.** Source: WHO [1] and G-FINDER [24].

It is now possible to estimate the RoI factor for national TB R&D programs, and by extrapolation to other indications, for all national health R&D. The results of the calculations for both Global Justice Index and RoI factor are shown in Figure 6; these recommended values are compared against actual values in Figure 7.

Based on this approach, the South African TB R&D allocation should be $92 million per annum, of which the bulk can be justified solely on the basis of the RoI Factor. Interestingly, the present TB expenditure of both the United States and the United Kingdom are already close to the recommended levels (90% and 94% of the calculated targets, respectively); however China, South Africa and Japan are below 1% of the target, and considerable increases in their respective TB R&D budgets are both required and justifiable.

## Conclusions

Although the moral and legal obligation (within the global justice framework) of high-income countries to the support of health research has been persuasively and extensively argued [26,27], no studies have been published on the extent of this obligation [22]. Moreover it can be argued that high-burden countries could realize a significant return on investment from neglected disease R&D through savings to public expenditure as a consequence of improved treatment regimens, diagnostics, vaccines and operations. The quantity of both this obligation and opportunity are the focus of this article. By considering the two separate concepts of global justice and return on investment, a methodology for calculating the scale of any country’s investment in TB R&D as a function of both per capita income and disease incidence has been defined.

The recent CEWG report concludes that “a binding instrument on R&D is necessary to secure appropriate funding and coordination to promote R&D needed to address the diseases that disproportionately affect developing countries and which constitute a common global responsibility” [22], and proposes a target of 0.01% of GDP for government-funded R&D be devoted to the health needs of developing countries, with little explanation of, or justification for, how the value was obtained. This paper goes some way towards defining how such a binding instrument could be constructed and proportioned at the level of a specific disease, and the extent of this responsibility for all countries.

It is not possible to directly compare the new target to the CEWG proposal, since the latter refers only to health needs in general and this study deals specifically with TB. However, the new target is mostly between 5% and 10% of the CEWG proposal, suggesting that relative to other priorities TB R&D should be a focus of neglected disease research in most countries, and especially high-burden countries.

## Abbreviations

CEWG: WHO’s Consultative Expert Working Group on R&D; CHRD: Commission on Health Research and Development; GERD: Gross expenditure on R&D; IPT: Isoniazid preventative therapy; Mtb: *Mycobacterium* tuberculosis; NSDA: Negotiated Service Delivery Agreement; rNPV: Risk-adjusted net present value; RoI: Return on investment factor; TB: Tuberculosis.

## Competing interests

The author declares that he has no competing interests.

## Supplementary Material

Additional file 1: Appendix A Data on TB treatment cost and disease burden in South Africa. **Appendix B.** Costs of TB Drug Development [36,37]. Click here for file
