# Supplementary material for: Determining quantitative targets for public funding of tuberculosis research and development
Source: Health Res Policy Syst. 2013 Mar 8;11:10. doi: 10.1186/1478-4505-11-10 (PMC3599983; doi:10.1186/1478-4505-11-10)
Supplement: Additional file 1: Appendix A — Data on TB treatment cost and disease burden in South Africa. Appendix B. Costs of TB Drug Development [36,37]. [file 1478-4505-11-10-S1.docx]

**Additional Material**

**Appendix A: Data on TB treatment cost and disease burden in South Africa**

INSERT TABLE 2 HERE

INSERT TABLE 3 HERE

**Appendix B: Costs of TB Drug Development**

The direct cost of new drug development, based on the process as shown in Table 4, has been extensively researched and reported. In a study published in 2003 [15], DiMasi presented data on average times and costs for new or investigational compounds as a function of different therapeutic areas.

INSERT TABLE 4 HERE

An alternative method to the estimation of drug costs can be estimated by dividing the actual R&D expenditure for a single company by the number of new drugs approved as shown in Table 5.

INSERT TABLE 5 HERE

Some work has also been completed specifically on TB drug costs; for instance a publication by the TB Alliance estimates the cost of developing a new TB drug at between $115 million to $240 million [12].

In this article, use is made of risk-adjusted net present (rNPV) value to calculate the cost. Evaluation of the rNPV requires a number of assumptions about the form of the public/private partnership and the nature of the market opportunity, including the following:

- the form of product or service under development (drug, vaccine, diagnostic, health service, etc); a case study of a novel TB drug has been used in the remainder of this section
- the point at which the private sector partner is financially involved in the project (and hence what proportion of the development costs)
- the project development costs as a function of stage (such as Phase I, II and III) and time (see the Appendix for more details of standard drug development costs)
- the market volume and value for the product or services under developed (e.g. one million patients treated per year at a cost of $28 per patient)
- the gross margin for the product (revenue less cost of manufacture and marketing)
- the product’s selling price (usually $/treatment cost).

The model assumes a market volume of 1·2 million patients at a treatment cost of $5·3 per patient. Peak revenues would be reached about 12 years after initiation of hit-to-lead optimisation, and would decline quickly after patent expiry (15 years from date of registration). The public sector licensee would receive an annual royalty of 10%, the discount rate applied is 8%, and a gross manufacturing margin of 50% would be realised. The success rates per stage as applied in the model were obtained from previously published data [14] [15] [37].

Table 2. South Africa’s Department of Health 2011/12 budget for TB treatment

| **Budget Item** | **Amount**  **(R million)** | **Amount**  **($ million)^*^** |
| --- | --- | --- |
| First line TB Drugs | 118 | 14 |
| MDR TB Drugs | 2,120 | 249 |
| TB Hospitalisation | 1, 60 | 207 |
| Management and Supervision of TB Treatment | 335 | 39 |
| TB Laboratory Support | 356 | 42 |
| Outpatient Visits | 276 | 32 |
| Other | 465 | 55 |
| **Total** | **4,965** | **584** |

*$1 = R8.5 (June 2012)

Source: DoH [3]

Table 3. TB statistics for South Africa (2010)

|  | **Total Number** | **Rate**  **(per 100,000 population)** |
| --- | --- | --- |
| Mortality (excluding HIV) | 25,000 | 50 |
| Mortality (including TB/HIV co-morbidity) | 48,000 | 96 |
| Prevalence | 400,000 | 795 |
| Incidence (including HIV) | 490,000 | 981 |
| Incidence (TB only) | 300,000 | 591 |
| HIV Incidence | 60% of TB patients are HIV positive | |
| Completion Rate | 73% of new smear positive cases | |
| Case Detection | 72% of total cases | |

Source: WHO [1]

Table 4. Stage costs and duration for investigational compounds

| **Phase** | **Costs**  **($ million)** | **Duration (years)** | **Success Rate** | **Adjusted Cost**  **($ million)** | **Cumulative Future Value**  **($ million)** |
| --- | --- | --- | --- | --- | --- |
| Hit Generation | 0·5 | 1 | 3% | 20 | 20 |
| Hit to Lead | 1 | 1 | 5% | 20 | 41·6 |
| Lead Optimisation | 2 | 1 | 8% | 25 | 69·9 |
| Preclinical | 5 | 2 | 10% | 50 | 131·6 |
| Phase I | 23 | 1 | 20% | 115 | 257·1 |
| Phase II | 20 | 2 | 30% | 66·7 | 366·5 |
| Phase III | 137 | 2 | 67% | 204·5 | 632·0 |
| Phase IV | 9 | 5 | 46% | 19·6 |  |
| Registration | 5 | 1 | 81% | 6·2 | 709·9 |
| **Total** | **202·5** | **16** | 100% | **526·9** | **709·9** |

Sources: DiMasi [15], Mathieu [14]

Table 5. Further Statistics on Drug Costs

| **Company** | **Number of drugs approved** | **R&D Spending Per Drug ($ mill)** | **Total R&D Spending 1997-2011 ($ mill)** |
| --- | --- | --- | --- |
| AstraZeneca | 5 | 11,791 | 58,955 |
| GlaxoSmithKline | 10 | 8,171 | 81,708 |
| Sanofi | 8 | 7,909 | 63,274 |
| Roche Holding AG | 11 | 7,804 | 85,841 |
| Pfizer Inc. | 14 | 7,727 | 108,178 |
| Johnson & Johnson | 15 | 5,886 | 88,285 |
| Eli Lilly & Co. | 11 | 4,577 | 50,347 |
| Abbott Laboratories | 8 | 4,496 | 35,970 |
| Merck & Co Inc | 16 | 4,210 | 67,360 |
| Bristol-Myers Squibb Co. | 11 | 4,152 | 45,675 |
| Novartis AG | 21 | 3,983 | 83,646 |
| Amgen Inc. | 9 | 3,692 | 33,229 |

Source: Herper [36]
